# PUS7‐dependent pseudouridylation of *ALKBH3* mRNA inhibits gastric cancer progression

**DOI:** 10.1002/ctm2.1811

**Published:** 2024-08-23

**Authors:** Yongxia Chang, Hao Jin, Yun Cui, Feng Yang, Kanghua Chen, Wenjun Kuang, Chunxiao Huo, Zhangqi Xu, Ya Li, Aifu Lin, Bo Yang, Wei Liu, Shanshan Xie, Tianhua Zhou

**Affiliations:** ^1^ Children's Hospital Zhejiang University School of Medicine National Clinical Research Center for Child Health Hangzhou Zhejiang China; ^2^ Department of Cell Biology Zhejiang University School of Medicine Hangzhou Zhejiang China; ^3^ Binjiang Institute of Zhejiang University Zhejiang University Hangzhou Zhejiang China; ^4^ International Institutes of Medicine the Fourth Affiliated Hospital of Zhejiang University School of Medicine Yiwu Zhejiang China; ^5^ MOE Laboratory of Biosystem Homeostasis and Protection College of Life Sciences Zhejiang University Hangzhou Zhejiang China; ^6^ Institute of Pharmacology and Toxicology Zhejiang Province Key Laboratory of Anti‐Cancer Drug Research College of Pharmaceutical Sciences Zhejiang University Hangzhou Zhejiang China; ^7^ School of Medicine Hangzhou City University Hangzhou Zhejiang China; ^8^ Metabolic Medicine Center International Institutes of Medicine and the Fourth Affiliated Hospital Zhejiang University School of Medicine Yiwu Zhejiang China; ^9^ Zhejiang University Cancer Center Hangzhou Zhejiang China

**Keywords:** alkbh3, gastric cancer, pseudouridylation, pus7

## Abstract

**Background:**

RNA pseudouridylation is a critical post‐transcriptional modification that influences gene expression and impacts various biological functions. Despite its significance, the role of mRNA pseudouridylation in cancer remains poorly understood. This study investigates the impact of pseudouridine synthase 7 (PUS7)‐mediated pseudouridylation of Alpha‐ketoglutarate‐dependent Dioxygenase alkB Homolog 3 (*ALKBH3*) mRNA in gastric cancer.

**Methods:**

Immunohistochemistry and Western blotting were used to assess PUS7 protein levels in human gastric cancer tissues. The relationship between PUS7 and gastric cancer progression was examined using 3D colony formation assays and subcutaneous xenograft models. Real‐time quantitative PCR (RT‐qPCR), Western blotting, and polysome profiling assays were conducted to investigate how PUS7 regulates ALKBH3. A locus‐specific pseudouridine (Ψ) detection assay was used to identify Ψ sites on *ALKBH3* mRNA.

**Results:**

Our findings indicate a significant reduction of PUS7 in gastric cancer tissues compared to adjacent non‐tumour tissues. Functional analyses reveal that PUS7 inhibits gastric cancer cell proliferation and tumour growth via its catalytic activity. Additionally, PUS7 enhances the translation efficiency of *ALKBH3* mRNA by modifying the U696 site with pseudouridine, thereby attenuating tumour growth. Importantly, ALKBH3 functions as a tumour suppressor in gastric cancer, with its expression closely correlated with PUS7 levels in tumour tissues.

**Conclusions:**

PUS7‐dependent pseudouridylation of *ALKBH3* mRNA enhances its translation, thereby suppressing gastric cancer progression. These findings highlight the potential significance of mRNA pseudouridylation in cancer biology and suggest a therapeutic target for gastric cancer.

**Highlights:**

- PUS7 enhances the translation efficiency of ALKBH3 through its pseudouridylation activity on ALKBH3 mRNA, thereby inhibiting gastric tumourigenesis.
- The expression levels of PUS7 and ALKBH3 are significantly correlated in gastric tumours, which may be potential prognostic predictors and therapeutic targets for patients with gastric cancer.

## Graphical abstract

1. PUS7 and ALKBH3 are characterized as novel tumour suppressors in gastric cancer.

2. PUS7 enhances the translation efficiency of ALKBH3 through its pseudouridylation activity on *ALKBH3* mRNA, thereby inhibiting gastric tumourigenesis.

3. The expression levels of PUS7 and ALKBH3 are significantly correlated in gastric tumours, which may be potential prognostic predictors and therapeutic targets for patients with gastric cancer.

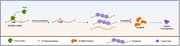

## 1 INTRODUCTION

Pseudouridylation is one of the most prevalent RNA modifications, playing indispensable roles in multiple cellular processes.^1^ RNA pseudouridylation can enhance base stacking, improve base pairing, and rigidify the sugar‐phosphate backbone, resulting in RNA structural changes that modulate gene expression.^2^ Previous studies have primarily focused on pseudouridine (Ψ) sites in tRNAs, rRNAs, and snRNAs, which are highly abundant and thus easier to identify biochemically.^3^, ^4^ Dysregulation of Ψ deposition in rRNAs and tRNAs has been linked to various biological processes, including tumourigenesis.^5^, ^6^, ^7^, ^8^ Recent advances in the transcriptome‐wide mapping of Ψ have revealed its presence in mRNA targets.^9^, ^10^, ^11^, ^12^ However, the biological function of mRNA pseudouridylation remains largely unknown.

Pseudouridylation is formed by base‐specific isomerization of uridine (U) catalysed by pseudouridine synthases (PUS).^2^, ^13^ PUS enzymes are classified into six families: TruA (tRNA pseudouridine synthase A), TruB, TruD, RsuA (ribosomal small subunit pseudouridine synthase A), RluA (23S rRNA and tRNA pseudouridine synthase) and Pus10 (pseudouridine synthase 10).^14^, ^15^ Among them, PUS7 is the only known member of the TruD family.^16^ PUS7‐mediated Ψ modification participates in various biological processes, including cancer development, by modifying Ψ in tRNAs.^7^, ^8^, ^17^ Inactivation of PUS7 impairs tRNA‐derived small fragment (tRF)‐mediated translation regulation in embryonic stem cells, resulting in increased protein biosynthesis and defective germ layer specification.^17^ PUS7 has been implicated in promoting the tumourigenesis of glioblastoma stem cells via PUS7‐dependent tRNA modification.^7^ PUS7 also facilitates the metastasis of colorectal cancer cells by upregulating LASP1 (LIM and SH3 protein 1), independent of PUS7's catalytic activity.^18^ Recent studies have discovered that PUS7 can modify Ψ in mRNAs in mammalian cells.^9^, ^12^ Despite this, the function of PUS7‐mediated mRNA pseudouridylation is still unclear.

ALKBH3 is a member of the alpha‐ketoglutarate‐dependent dioxygenase AlkB homology family, which can remove alkyl adducts from nucleobases by oxidative dealkylation.^19^ Previous studies have shown that ALKBH3 is an alkylation damage repair enzyme, repairing m^1^A and m^3^C (3‐methylcytosine) alkylation damage in RNA and ssDNA.^20^ and playing an oncogenic role in various cancers.^21^ For instance, ALKBH3 can demethylate the m^1^A on tRNA, making tRNA more sensitive to angiogenin (ANG) cleavage, thereby generating tRNA‐derived small RNAs (tDRs) around the anticodon regions and promoting cancer cell proliferation, migration and invasion.^22^ Additionally, ALKBH3 has been found to improve the translation efficiency by demethylating m^1^A on *CSF‐1* (colony stimulating factor 1) mRNA, enhancing the aggressiveness of ovarian and breast cancer cells.^23^ However, the role of ALKBH3 in gastric cancer progression has not been reported.

Here, our study reveals that the expression of PUS7 and ALKBH3 are both significantly reduced in gastric cancer tissues. PUS7 and ALKBH3 exert an inhibitory effect on the proliferation and tumour growth of gastric cancer cells. Importantly, PUS7 functions as a gastric tumour suppressor gene through its catalytic activity. Mechanically, PUS7 modifies *ALKBH3* mRNA with Ψ, increasing its translation efficiency and thereby suppressing gastric tumour growth. Thus, we identify PUS7 and ALKBH3 as novel gastric cancer suppressor genes, and our findings suggest that PUS7‐dependent pseudouridylation of *ALKBH3* mRNA inhibits gastric carcinogenesis.

## 2 MATERIALS AND METHODS

### 2.1 Human tissue collection and characterization

Two cohorts of patients with gastric cancer were enrolled, and gastric tumour tissues and corresponding non‐tumour tissues were collected. All participants have provided written informed consent. Tissue microarray samples (cohort 1) were obtained from the Second Affiliated Hospital, Zhejiang University School of Medicine (Hangzhou, China). Cohort 2 included 52 pairs of fresh gastric tumour tissues and their pair‐matched non‐tumour tissues and was obtained from the First Affiliated Hospital, Zhejiang University School of Medicine (Hangzhou, China).

Gastric tumour tissue was excised from the central part of the tumour through surgical resection. The pair‐matched non‐tumour tissue was taken from normal tissue located more than 5 cm from the edge of the cancer and confirmed to have a negative margin. The study was approved by the Ethics Committee Review Board of Zhejiang University School of Medicine.

### 2.2 Animals

Mouse experiments were conducted in accordance with the Guide for the Care and Use of Animals for research purposes and were approved by the Committee of Animal Ethics, Zhejiang University. Four‐week‐old female nude mice were bred in a specific pathogen‐free environment in the Animal Facility, Zhejiang University. Cells were trypsinized, washed and resuspended in PBS/Matrigel (1:1) and subcutaneously injected into the flank of nude mice (5 × 10^6^ cells per flank of mouse). The tumours were dissected and weighed at the endpoint. Tumour diameters were measured with callipers, and tumour volumes were calculated using the formula *V* = (*L* × *W* ^2^)/2, where *V* = volume (in cm^3^), *L* = length (in cm), *W* = width (in cm).

### 2.3 Cell culture

AGS, HGC‐27 and SNU‐1 cells were obtained from the Cell Bank of Type Culture Collection of Chinese Academy of Sciences (Shanghai, China). NCI‐N87 and MKN45 cells were acquired from the American Type Culture Collection (ATCC). All gastric cancer cells were cultured in Roswell Park Memorial Institute (RPMI) 1640 (Corning) medium supplemented with 10% fetal bovine serum (ExCell). HEK293T cells were acquired from the ATCC and cultured in Dulbecco's Modification of Eagle's Medium (Corning) supplemented with 10% fetal bovine serum. All cells were maintained in a humidified atmosphere at 37°C with 5% CO_2_.

### 2.4 Immunohistochemistry

Gastric cancer tissue array (cohort 1) was subjected to immunohistochemical staining with anti‐PUS7 or ALKBH3 antibody following a previously reported protocol with minor modifications.^24^ The gastric cancer tissue array was stained using anti‐PUS7 or anti‐ALKBH3 antibody (1:100) and incubated for 1 h. Horseradish‐peroxidase‐labelled secondary antibody was incubated for 30 min, and coloured by DAB kit (Invitrogen). The tissue array was then counterstained with haematoxylin and analyzed with a Panoramic MIDI tissue array scanner (3D HISTECH). The expression levels of PUS7 and ALKBH3 were determined by immunohistochemistry score (H‐score) based on the positive rate and staining intensity. H‐score was acquired according to the formula: H‐score  =  (percentage of cells of weak intensity × 1) + (percentage of cells of moderate intensity × 2) + (percentage of cells of strong intensity × 3). The following primary antibodies were used: rabbit anti‐PUS7 (HPA024116, Atlas Antibodies) and rabbit anti‐ALKBH3 (12292‐1‐AP, Proteintech).

### 2.5 Plasmids

Human *PUS7* and *ALKBH3* mRNAs were amplified from HEK293T cell cDNA using Phanta Max Master Mix (Vazyme) and separately inserted into the *BamH* I and *Xho* I/ *BamH* I and *EcoR* I restriction enzyme sites in the pLVX‐puro vectors (Addgene). To generate the PUS7‐D294A mutant, the wild‐type PUS7 plasmid was used as a template. For rescue experiments, PUS7, PUS7‐D294A and ALKBH3 mutants resistant to shRNAs were produced using PCR and cloned into pLVX‐puro vectors. To stably knock down PUS7 and ALKBH3, shRNA oligos were inserted into the *Age* I and *EcoR* I restriction enzyme sites in the pLKO.1 vector (Addgene). shPUS7‐1, 5′‐CCGGTCTTAGTTCAGACTCATATATCTCGAGATATATGAGTCTGAACTAAGATTTTTG‐3′; shPUS7‐2, 5′‐ CCGGGGCACTGGTTGTCGAAGATAACTCGAGTTATCTTCGACAACCAGTGCCTTTTTG‐3′; shALKBH3‐1, 5′‐ CCGGCCCATTATTGCTTCACTAAGTCTCGAGACTTAGTGAAGCAATAATGGGTTTTTTG‐3′; shALKBH3‐2, 5′‐CCGGTATCAGCAACCAAGACTTACACTCGAGTGTAAGTCTTGGTTGCTGATATTTTTTG‐3′.

To construct the CRISPR/Cas9 plasmids, the synthesized sgRNAs were annealed in a thermal cycler and then cloned into the lentiCRISPR V2 vector (Addgene) after linearisation with *BsmBI* digestion. All constructs were confirmed by Sanger sequencing. The primers used for the plasmids are listed in Table S1, and the sequences of sgRNAs targeting the *PUS7* or *ALKBH3* gene are listed in Table S2.

### 2.6 Lentivirus infection

Lentiviruses were produced by co‐transfecting pCMV‐δ8.91 (Addgene), VSV‐G (Addgene) and the indicated vectors (pLVX‐PUS7, pLVX‐PUS7‐D294A or pLVX‐ALKBH3) in HEK293T cells using PolyJet (SignaGen). The medium was replaced 12 h after transfection, and cells were incubated for 48 h for virus production. The supernatant containing PUS7‐ or ALKBH3‐expressing lentiviruses was collected, and cell debris was removed with a 0.22 µm filter unit (Millipore). AGS or MKN45 cells cultured in 6‐well plates were infected with 1 mL of virus stock immediately for 24 h. Cells were then selected with puromycin for an additional 24 h and subjected to further experiments.

### 2.7 Transfection

The small interfering RNA (siRNA) oligonucleotides were transfected into MKN45 by Lipofectamine RNAiMAX (Invitrogen) according to the manufacturer's instructions. Cells were incubated for 48 h and subjected to further experiments. sinc, UUCUCCGAACGUGUCACGUTT; siALKBH3‐1, GAAAGAAGCUGACUGGAUA; siALKBH3‐2, ATCGCTATCATCTTTAGGCAA; siPUS7‐1, GCUAGGGAAUUUCAGCUAUTT; siPUS7‐2, GGAAUAACAUGGUAAGCAATT.

### 2.8 Western blotting

Protein from each cell line was extracted using RIPA buffer (50 mM Tris (pH 7.5), 0.1% SDS, 1% NP40, 0.5% deoxycholate, 150 mM NaCl) supplemented with a complete protease inhibitor cocktail (Roche). Cell lysates were sonicated and centrifuged at 10 000 × *g* for 5 min at 4°C. Protein concentration was measured using a Pierce BCA protein assay kit (Thermo Scientific). Protein samples were separated by 10% sodium dodecyl sulphate‐polyacrylamide gel electrophoresis (SDS‐PAGE) and transferred onto a polyvinylidene fluoride (PVDF) membrane (BioRad). Detection was performed using an ECL chemiluminescence kit (Fdbio science). Images were captured using the ChemiDoc Touch Imaging System (Bio‐Rad). The primary antibodies used were rabbit anti‐PUS7 (HPA024116, Atlas Antibodies), rabbit anti‐ALKBH3 (ab227496, Abcam), and rabbit anti‐β‐actin (AC026, Abclonal). All primary antibodies were used at a 1:2000 dilution in 5% skim milk, and all secondary antibodies were used at a 1:5000 dilution in 5% skim milk. β‐actin was used as a loading control.

### 2.9 RNA isolation and RT‐qPCR

Total RNAs were extracted using TRIzol Reagent (Invitrogen) according to the manufacturer's instructions. cDNA was synthesized using the High‐Capacity cDNA Reverse Transcription Kit (Applied Biosystems). Reverse transcription was performed at 37°C for 15 min, followed by enzyme inactivation at 95°C for 5 min using a PCR machine. Primer sets were purchased from Shanghai Shangya Biotechnology and used at a final concentration of 0.2 mM. Real‐time quantitative PCR (RT‐qPCR) was performed on the 480 II Real‐Time PCR System (Roche) using the ChamQ Universal SYBR qPCR Master Mix (Vazyme) following the manufacturer's protocol. All RT‐qPCR assays were performed using at least three independent experiments with three different samples. Target gene expression levels were normalized against that of the *snRNA U6* gene, and relative expression was calculated using the 2^−△△Ct^ method. The primers (PUS7 1#, ALKBH3 1#, U6 1#) used for RT‐qPCR are listed in Table S3.

### 2.10 3D colony formation assay

Matrigel matrix (Corning) was mixed with serum‐free RPMI‐1640 medium in a ratio of 1:10 and added to a 96‐well plate, followed by incubation at 37°C for at least 60 min. The upper layer of non‐solidified liquid was removed, and 5 × 10^3^ transfected cells were mixed with the Matrigel matrix in the same volume and seeded into each well. After being cultured in the incubator for 15 min, RPMI‐1640 medium was added above the cell‐gel mix for colony formation. After 7−10 days, the morphology of colonies was captured with a 10× lens on a confocal microscope (OLYMPUS FV1000‐IX81), and the number of colonies was determined using Image J software (NIH).

### 2.11 Migration assay

A total of 5 × 10^4^ cells in culture medium containing 1% fetal bovine serum were seeded in the upper chambers of transwell inserts (8‐µm pore, 3422, Corning). The lower chambers were filled with a culture medium containing 10% fetal bovine serum. After 25 h (for MKN45 cells), the migrated cells were stained with crystal violet, imaged using a 10× lens on a confocal microscope (OLYMPUS FV1000‐IX81), and quantified using Image J software (NIH).

### 2.12 Invasion assay

Transwell inserts (8‐µm pore, 3422, Corning) were precoated with 100 µL of a Matrigel (354234, Corning) and RPMI‐1640 medium mixture at a 1:10 ratio for 2 h at 37°C. A total of 5 × 10^4^ cells in a culture medium containing 1% fetal bovine serum were seeded in the upper chambers. The lower chambers were filled with a culture medium containing 10% fetal bovine serum. After 49 h (for MKN45 cells), the invaded cells were stained with crystal violet, imaged using a 10× lens on a confocal microscope (OLYMPUS FV1000‐IX81), and quantified using Image J software (NIH).

### 2.13 Locus‐specific Ψ detection with CMC

Locus‐specific Ψ detection assays were conducted as described previously.^25^ Total RNAs (10 µg) were fragmented into ∼150–200 nt fragments at 94°C for 2−3 min using magnesium RNA fragmentation buffer (New England Biolabs), followed by ethanol precipitation. The fragmented RNAs were resuspended, denatured in 20 µL 5 mM EDTA at 80°C for 5 min, and cooled on ice. For N‐cyclohexyl‐N′‐(2‐morpholinoethyl) carbodiimide (CMC) labelling, 10 µL of denatured RNA was added to 100 µL of BEU buffer (50 mM Bicine (pH 8.5), 4 mM EDTA, 7 M urea) containing 0.2 M CMC (Sigma) as the ‘+ CMC’ sample or added to 100 µL BEU buffer without CMC as the ‘‐ CMC’ sample. The CMC reaction was carried out at 37°C for 20 min, followed by ethanol precipitation to remove remaining CMC. RNAs recovered from the ‘+ CMC’ and ‘‐ CMC’ samples were next separately dissolved in 50 µL of Na_2_CO_3_ buffer (50 mM sodium carbonate (pH 10.4), 2 mM EDTA), and incubated at 37°C for 6 h. Another ethanol precipitation step was performed to recover RNAs, and RNAs were dissolved in 10 µL of nuclease‐free water. The samples were added to 1 µL of 100 µM random hexamer primer (TaKaRa) and incubated at 65°C for 5 min, followed by chilling on ice. The annealed RNAs were added to 8 µL of freshly prepared reverse transcription buffer (125 mM Tris (pH 8.0), 15 mM MnCl_2_, 187.5 mM KCl, 1.25 mM dNTPs, 25 mM DTT) to a final Mn^2+^ concentration of 6 mM, and incubated at 25°C for 2 min. Reverse transcription was performed by adding 1 µL of SuperScript II reverse transcriptase (Invitrogen) and incubating at 25°C for 10 min, 42°C for 3 h, and heat‐inactivated at 70°C for 15 min. The samples were then subjected to RT‐qPCR analysis using LightCycler 480 software (Roche). The primers (ALKBH3 2#) used for RT‐qPCR are listed in Table S3.

### 2.14 TA cloning assay

*ALKBH3* fragments were amplified by PCR. The PCR products were individually cloned in linearised pEASY‐Blunt Zero backbone (TransGen Biotech, China) using Taq DNA polymerase. 1 µL of pEASY‐Blunt Zero Cloning Vector to was added to 0.5–4 µL PCR product, followed by being mixed well and incubated at room temperature for 10 min for connection reaction. The connected product was mixed with 50 µL Trans1‐T1 competent cells, ice‐incubated for 20−30 min. Subsequently heat shocked at 42°C for 30 s, and then placed it on ice. The mixture was inoculated on the surface of freshly prepared LB (Luria‐Bertani) plate. After 18−20 h of incubation at 37°C, selected monoclonal colonies and verified by Sanger sequencing.

### 2.15 Polysome profiling

Polysome profiling was performed as previously described.^26^ Briefly, stable cells were treated with 100 µg/mL cycloheximide (MedChemExpress) for 5 min at 37°C, followed by washing twice with ice‐cold phosphate‐buffered saline (PBS) containing 100 µg/mL cycloheximide. Cells were lysed using 700 µL polysome lysis buffer (15 mM Tris‐HCl, 100 mM KCl, 5 mM MgCl_2_, 2 mM DTT (dithiothreitol), 1% Triton X‐100, 1 mg/mL heparin sodium, 100 µg/mL cycloheximide). Cell lysates were incubated on ice for 10 min and centrifuged at 13 000 rpm at 4°C for 10 min. The supernatant was kept, and its absorbance was measured at 260 nm. Then 500 µL supernatant was loaded onto 11 mL of 5%−50% sucrose gradient (15 mM Tris‐HCl, 100 mM KCl, 5 mM MgCl_2_, 2 mM DTT, 1 mg/mL heparin sodium, 100 µg/mL cycloheximide) and centrifuged at 36 000 rpm in a Beckman SW‐41Ti rotor for 2 h at 4°C. The gradients were fractionated and monitored at an absorbance 254 nm (Brandel). Polysome‐associated and cytosolic RNA were isolated using TRIzol (Invitrogen) and subjected to RT‐qPCR analysis.

### 2.16 Quantification and statistical analysis

For each experiment, sample size, number of replicates, and associated statistical data can be found in the results section and figure legends and/or the respective Materials and methods section. Data are representative of at least three independent experiments. Pearson correlation analysis was used to determine the correlation between PUS7 and ALKBH3 levels in gastric tumour tissues. *p* values were calculated using two‐sided Student's *t*‐tests on GraphPad Prism 7. Statistical significance was defined as *p *< .05.

## 3 RESULTS

### 3.1 PUS7 is significantly downregulated in gastric tumour tissues

**FIGURE 1:**
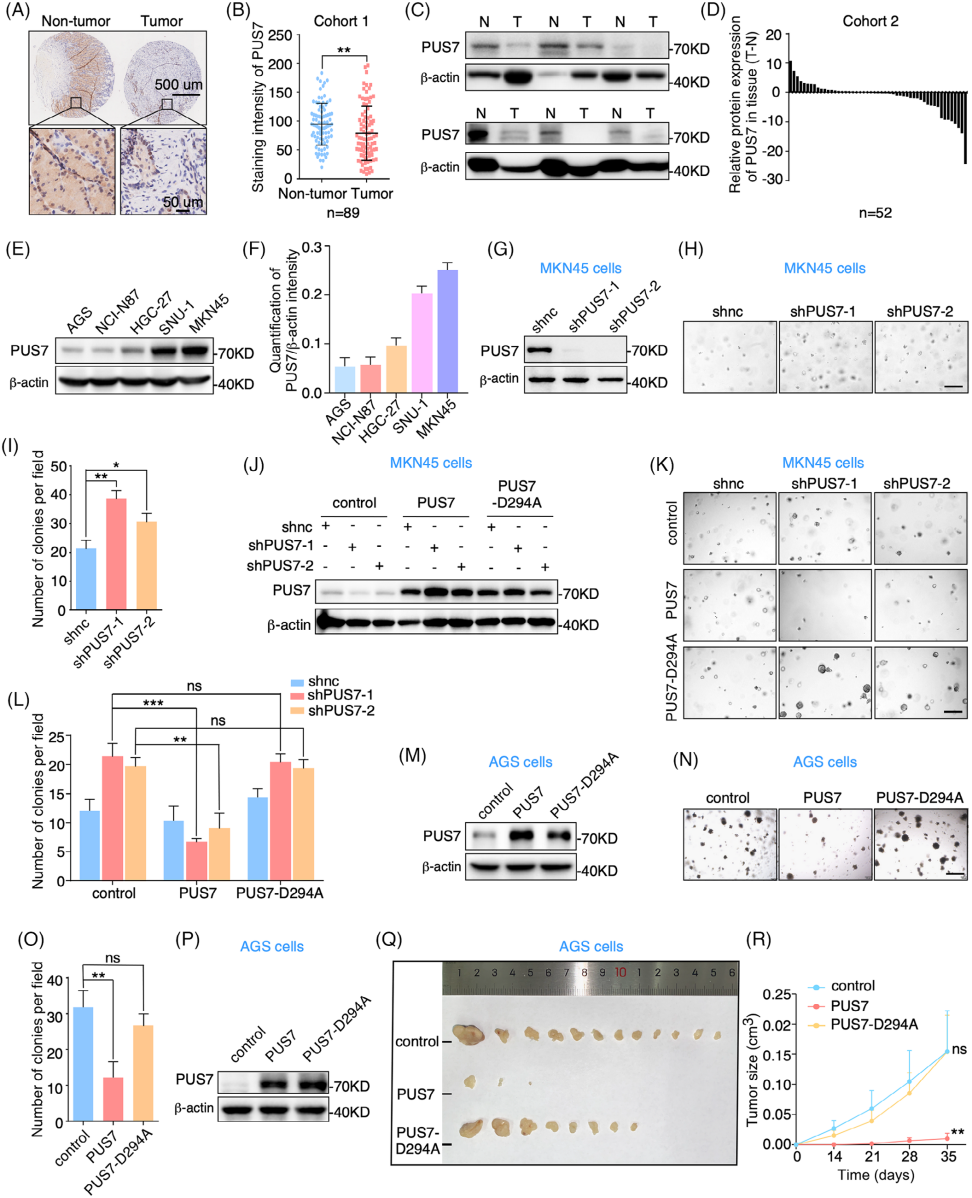
PUS7 is significantly downregulated in gastric tumour tissues and inhibits cancer cell proliferation. (**A, B**) Immunohistochemistry analysis with anti‐PUS7 antibody of gastric cancer tissue array from cohort 1. Relative PUS7 expression was analyzed. (**C, D**) Western blots of gastric tumour tissues and their corresponding non‐tumour tissues with anti‐PUS7 and β‐actin antibodies. β‐actin, a loading control. N, non‐tumour; T, tumour. Relative levels of PUS7 are shown. (**E, F**) Western blotting with anti‐PUS7 and β‐actin antibodies in various gastric cancer cell lines. β‐actin, a loading control. The intensity of PUS7 was measured based on the normalization to β‐actin (F). (**G**–**I**) MKN45 cells infected with lentivirus‐carrying shRNAs targeting *PUS7* (shPUS7‐1 or shPUS7‐2) were subjected to western blots and 3D colony formation assays. Quantification of colony number per field is presented. Scale bar, 200 µm. shnc, negative control shRNA. (**J**–**L**) MKN45 cells treated with the indicated shRNAs were infected with lentivirus expressing PUS7 or PUS7‐D294A plasmid and subjected to western blots and 3D colony formation analyses. The colony number was analyzed. Scale bar, 200 µm. (**M**‐**O**) AGS cells were infected with lentivirus expressing PUS7 or PUS7‐D294A plasmid and applied for western blots and 3D colony formation assays. Quantification of colony number per field is shown. Scale bar, 200 µm. (**P**‐**R**) AGS cells infected with lentivirus expressing the indicated plasmids were processed to subcutaneous implantation in BALB/C nude mice. The gloss morphology of subcutaneous tumours (Q) and tumour growth curve (R) were shown. Data are expressed as means   ± SD. Student's *t*‐test (tumour growth data (R) are expressed as means ± SEM. Two‐way ANOVA); *p < .05, **p < .01, ***p < .001, ns, not significant.

To investigate the role of PUS7 in gastric cancer, we examined the protein expression of PUS7 in human gastric tumour tissues from two independent cohorts. Immunohistochemical staining of the tissue microarray revealed that the levels of PUS7 protein were significantly downregulated in gastric tumour tissues compared with their corresponding non‐tumour tissues from cohort 1 (Figures 1A and B and S1). In cohort 2, immunoblotting of gastric tumour tissues and their paired non‐tumour tissues confirmed that PUS7 protein levels were also obviously reduced in gastric tumour tissues (Figure 1C and D). These data suggest that PUS7 may have an important function in gastric carcinogenesis.

### 3.2 PUS7 inhibits gastric cancer cell proliferation via its Ψ synthase activity

To determine the function of PUS7 in gastric carcinogenesis, we first detected the level of PUS7 protein in human gastric cancer cell lines. Our results showed that PUS7 was highly expressed in MKN45 cells but lowly expressed in AGS cells (Figure 1E and F). Since three‐dimensional (3D) cell culture is more biologically relevant to living organisms than two‐dimensional culture,^27^ we employed 3D colony formation assays to detect the effect of PUS7 on gastric cancer cell proliferation. Our data revealed that depletion of PUS7 with short hairpin RNAs (shRNAs) significantly increases the colony formation ability in MKN45 cells (Figure 1G–I). Rescue experiments displayed that ectopic expression of PUS7 significantly attenuated the enhanced proliferation capacity of PUS7‐depleted MKN45 cells (Figure S2). Further migration and invasion assays revealed that depletion of PUS7 with small interfering RNA (siRNA) significantly increased the migration and invasion abilities of MKN45 cells (Figure S3A–D). Meanwhile, ectopic expression of PUS7 restored the increased migration and invasion phenotype in PUS7‐deficient cells (Figure S3E–H). These findings suggest that PUS7 acts as a tumour suppressor gene to inhibit the proliferation, migration and invasion of gastric cancer.

To address whether the role of PUS7 in gastric cancer cell growth is dependent on its Ψ synthase activity, we constructed a catalytically inactive mutant of PUS7 (PUS7‐D294A).^17^ The increased proliferation phenotype in PUS7‐depleted cells was significantly restored by ectopic expression of wild‐type PUS7, but not the PUS7‐D294A mutant (Figure 1J–L). In addition, overexpression of wild‐type PUS7 significantly reduced the colony number of AGS cells, whereas the PUS7‐D294A mutant had no significant effect on the proliferation of AGS cells (Figure 1 M–O). More importantly, subcutaneous implantation analysis of gastric cancer cells in nude mice revealed that overexpression of wild‐type PUS7, but not the PUS7‐D294A mutant, significantly suppressed the tumourigenicity of AGS cells (Figure 1P–R). Taken together, these findings indicate that PUS7 inhibits the proliferation and tumour growth of gastric cancer cells, which is dependent on its catalytic activity.

### 3.3 PUS7 increases ALKBH3 levels and its mRNA translation

**FIGURE 2:**
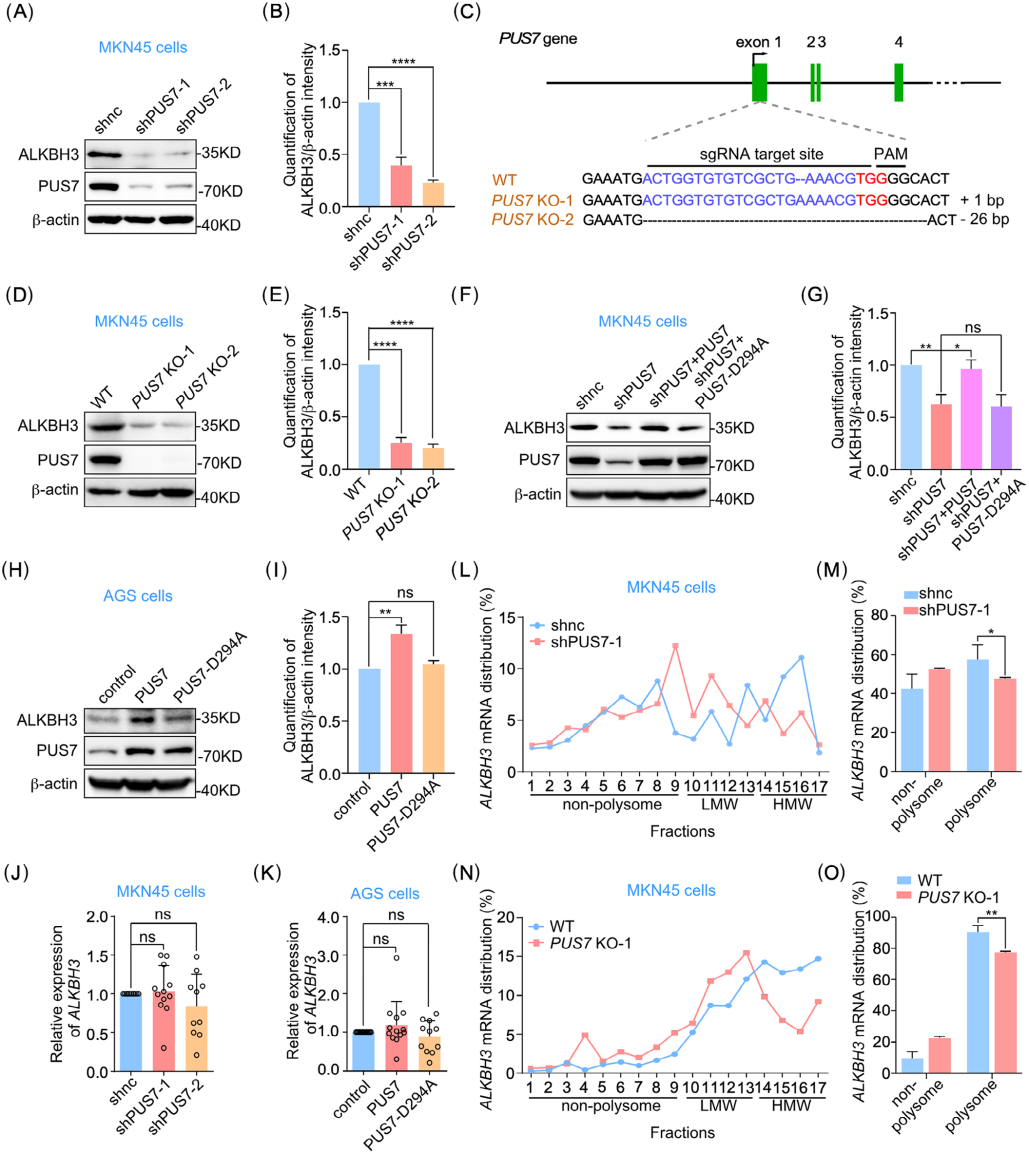
PUS7 increases the expression of ALKBH3 protein and the translation efficiency of *ALKBH3* mRNA. (**A, B**) Western blotting with anti‐ALKBH3 and PUS7 antibodies in MKN45 cells treated with the indicated shRNAs. β‐actin served as a loading control. The intensity of ALKBH3 was measured based on the normalization to β‐actin (B). (**C**) The sequence of indels in the *PUS7* locus of MKN45 cells generated by the CRISPR/Cas9 system is shown. The sgRNAs target site and the protospacer adjacent motif (PAM) site are indicated in blue and red, respectively. (**D, E**) Wild‐type (WT) or *PUS7* knockout (KO) MKN45 cells were subjected to western blots with the indicated antibodies. β‐actin, a loading control. The intensity of ALKBH3 was measured based on the normalization to β‐actin (E). (**F–I**) MKN45 or AGS cells treated with the indicated shRNAs or plasmids were applied for western blots with the antibodies as shown. β‐actin, a loading control. The intensity of ALKBH3 was measured based on the normalization to β‐actin (G, I). (**J**) MKN45 cells infected with lentivirus‐carrying shRNAs targeting *PUS7* (shPUS7‐1 or shPUS7‐2) were processed for RT‐qPCR analysis. *snRNA U6* was used as an internal control. (**K**) AGS cells infected with lentivirus expressing PUS7 or PUS7‐D294A were subjected to RT‐qPCR analysis. *snRNA U6*, an internal control. (**L, M**) MKN45 cells treated with shPUS7‐1 were lysed and subjected to sucrose gradient centrifugation and polysome profiling. The distribution of *ALKBH3* mRNA in different fractions is shown (L). And *ALKBH3* mRNA distribution in the fractions of non‐polysome and polysome was analyzed (M). (**N, O**) Wild‐type or *PUS7* knockout MKN45 cells were lysed and subjected to sucrose gradient centrifugation and polysome profiling. The distribution of *ALKBH3* mRNA in different fractions is shown (N). And *ALKBH3* mRNA distribution in the fractions of non‐polysome and polysome was analyzed (O). LMW, low molecular weight; HMW, high molecular weight. Data are shown as mean ± SD. Student's *t‐*test; *p < .05, **p < .01, ***p < .001, ****p < .0001, ns, not significant.

While studying the effect of PUS7 on gastric cancer cells, we made the serendipitous observation that knockdown of PUS7 dramatically reduced the expression of the demethylase ALKBH3 in MKN45 cells (Figure 2A and B). To confirm the regulation of ALKBH3 expression by PUS7, we constructed two *PUS7* knockout cell lines using the CRISPR/Cas9 technique (Figure 2C) and found that deletion of *PUS7* also robustly suppressed ALKBH3 expression (Figure 2D and E). To define if the expression of ALKBH3 is controlled by PUS7‐mediated pseudouridylation, we ectopically expressed wild‐type PUS7 or the PUS7‐D294A mutant in cells depleted of PUS7. Our data showed that exogenous expression of wild‐type PUS7, but not PUS7‐D294A, visibly rescued ALKBH3 levels in PUS7‐depleted MKN45 cells (Figure 2F and G). Furthermore, ectopic expression of wild‐type PUS7 rather than PUS7‐D294A obviously increased the expression of ALKBH3 in AGS cells (Figure 2H and I).

To explore how PUS7 regulates the protein expression of ALKBH3, we initially examined the levels of *ALKBH3* mRNA in PUS7‐depleted MKN45 cells and PUS7‐overexpressed AGS cells. The results showed that interfering with PUS7 expression had no significant effect on *ALKBH3* mRNA levels in both cells (Figure 2J and K). Thus, we continued to investigate whether PUS7 regulates the translation efficiency of *ALKBH3* mRNA. Polysome profile analysis revealed that more *ALKBH3* mRNA was present in the non‐polysome and low molecular weight fractions in PUS7‐depleted MKN45 cells compared with that of control cells (Figure 2L and M). Knockout of *PUS7* also promotes the shift of *ALKBH3* mRNA distribution to non‐polysome (Figure 2N and O), indicating that PUS7 depletion inhibits the translation efficiency of *ALKBH3* mRNA. Collectively, these data suggest that PUS7 increases ALKBH3 protein levels by facilitating its translation efficiency.

### 3.4 PUS7 decorates *ALKBH3* mRNA on the U696 site with Ψ

**FIGURE 3:**
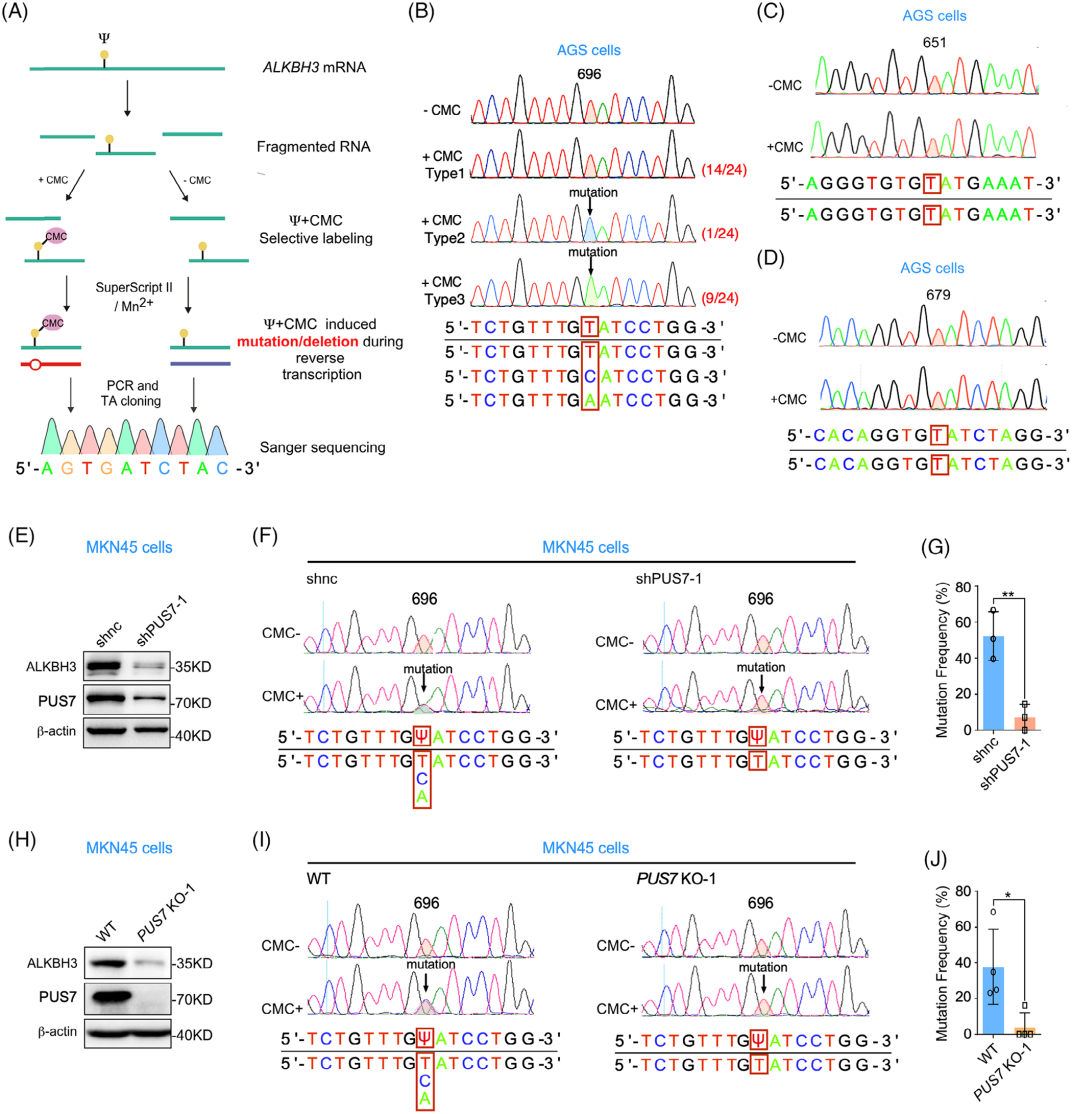
PUS7 decorates *ALKBH3* mRNA on U696 with Ψ. (**A**) Workflow of the method to detect the locus‐specific Ψ sites. (**B**–**D**) The PCR products of *ALKBH3* were subjected to TA cloning and Sanger sequencing. The results of three putative Ψ‐consensus sequences of *ALKBH3* mRNA from TA colonies were analyzed. (**E**–**G**) MKN45 cells infected with lentivirus‐carrying shRNAs targeting *PUS7* (shPUS7‐1) were subjected to immunoblotting and locus‐specific Ψ sites detecting. The results of U696 site mutation frequency of *ALKBH3* were analysed. (**H**–**J**) Wild‐type or *PUS7* knockout MKN45 cells were subjected to western blotting and locus‐specific Ψ sites detection. The results of U696 site mutation frequency of *ALKBH3* were measured. Percentage represents the mutation frequency calculated by taking the peak area of ‘C’ and ‘A’ peaks over the sum of ‘T’, ‘C’ and ‘A’ peaks (G and J). Ψ, pseudouridine; CMC, N‐cyclohexyl‐N′‐(2‐morpholinoethyl) carbodiimide. Data are shown as mean ± SD. Student's *t*‐test; *p < .05, **p < .01.

To identify Ψ sites on *ALKBH3* mRNA modified by PUS7, we analyzed the PUS7 core consensus sequence for pseudouridylation (UGUA, underline for Ψ site) within *ALKBH3* mRNA^9^, ^28^, ^29^ and found three potential sites (U651, U679 and U696) (Figure S4). Ψ‐containing RNAs can be specifically labelled by N‐cyclohexyl‐N′‐β‐(4‐methylmorpholinium) ethylcarbodiimide p‐tosylate (CMC) under alkaline conditions, and the Ψ‐CMC adduct generates a stop signature during reverse transcription by SuperScript III^9^, ^12^, ^28^, ^29^, ^30^ or introduces mutations at or around the Ψ sites in the synthesized cDNA during reverse transcription by SuperScript II.^25^ Based on these principles, we carried out CMC‐based RT with quantitative PCR (RT‐qPCR) assay^12^ to determine the Ψ sites on *ALKBH3* mRNA. The analysis displayed a notably reduced readthrough ratio at potential Ψ sites (contains U651, U679 and U696) on *ALKBH3* cDNA reverse transcription by SuperScript III, after CMC‐treatment and normalization to control regions (Figure S5). This experiment suggests the existence of Ψ in the potential sites of *ALKBH3* mRNA. We further used SuperScript II to validate the Ψ sites on *ALKBH3* mRNA after CMC treatment (Figure 3A). TA cloning and Sanger sequencing revealed the mutation of T to C/A at the 696 site of *ALKBH3* cDNA in AGS cells (Figure 3B). Meanwhile, no mutation was detected at the 651 and 679 sites of *ALKBH3* cDNA (Figure 3C and D). Moreover, depletion or knockout of PUS7 significantly decreased the mutation frequency at the 696 site of *ALKBH3* cDNA (Figure 3E–J). Together, these results indicate that PUS7 catalyses the U696 site of *ALKBH3* mRNA with Ψ.

### 3.5 PUS7 inhibits gastric tumour growth through ALKBH3

**FIGURE 4:**
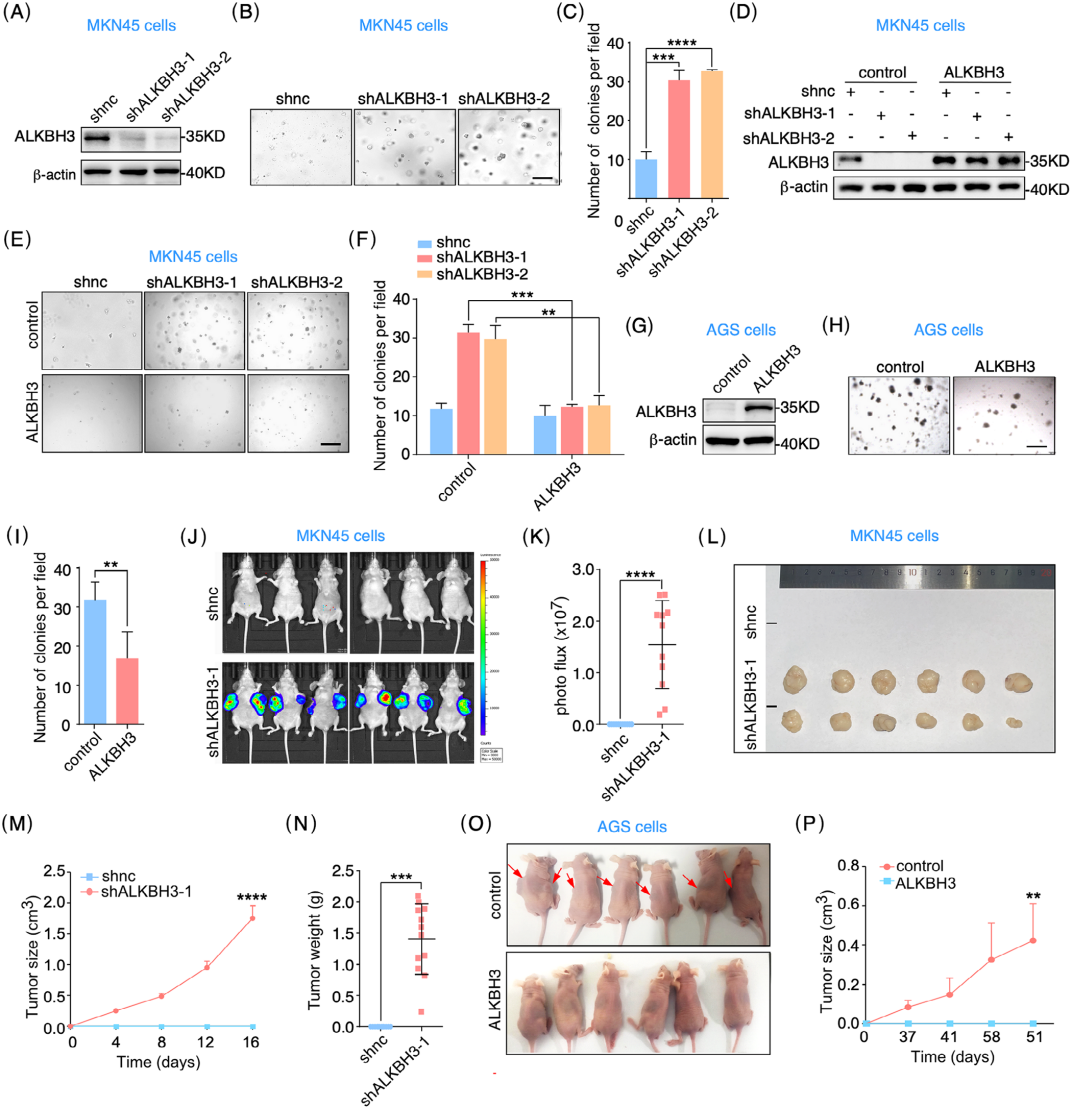
ALKBH3 suppresses gastric cancer cell proliferation and tumour growth. (**A**–**C**) MKN45 cells infected with lentivirus‐carrying shRNAs targeting *ALKBH3* (shALKBH3‐1 or shALKBH3‐2) were subjected to western blots and 3D colony formation assays. Quantification of colony number per field is presented. Scale bar, 200 µm. (**D**‐**F**) MKN45 cells treated with the indicated shRNAs were infected with the lentivirus expressing ALKBH3, and applied for western blots and 3D colony formation analyses. Analysis of colony number per field is shown. Scale bar, 200 µm. (**G**–**I**) AGS cells infected with lentivirus expressing ALKBH3 were processed to western blots and 3D colony formation assays. The colony number per field was calculated. (**J**–**N**) MKN45 cells infected with lentivirus targeting *ALKBH3* were subcutaneously injected into BALB/c nude mice. BLI (bioluminescence imaging) analysis was performed to measure tumour growth (J, K). The tumour growth curve (M) and weight (N) were analyzed. (**O**, **P**) AGS cells infected with lentivirus expressing ALKBH3 were applied for subcutaneous implantation in BALB/C nude mice. Arrows indicate tumours. Tumour growth curve is shown (P). Scale bars, 200 µm. Data are expressed as means   ± SD. Student's *t*‐test (tumour growth data (M, P) are expressed as means ± SEM. Two‐way ANOVA); *p < .05, **p < .01, ***p < .001, ****p < .0001.

Given that PUS7 suppresses gastric tumour growth and pseudouridylates *ALKBH3* mRNA, it is reasonable to hypothesize that ALKBH3 may be involved in the regulation of PUS7 in gastric carcinogenesis. Our 3D colony formation, migration, and invasion assays showed that the depletion of ALKBH3 significantly enhanced the proliferation and migration and invasion abilities of MKN45 cells (Figures 4A–C and S6A–D), which was significantly reversed by ectopic expression of ALKBH3 (Figures 4D–F and S6E–I). Knockout of *ALKBH3* had a similar phenotype to that of ALKBH3‐depleted cells (Figure S7). In addition, overexpression of ALKBH3 significantly suppressed the proliferation of AGS cells (Figure 4G–I). When MKN45 cells were subcutaneously injected into nude mice, our bioluminescence imaging (BLI) experiments revealed that knockdown of ALKBH3 significantly increased BLI signals compared with that of control cells (Figure 4J and K). ALKBH3 depletion also caused dramatic increases in the size and weight of subcutaneous tumours (Figure 4L–N). Moreover, ALKBH3 overexpression suppressed the growth of gastric tumours in a xenograft mouse model (Figure 4O and P). Taken together, these results suggest that ALKBH3 functions as a tumour suppressor gene in gastric cancer.

**FIGURE 5:**
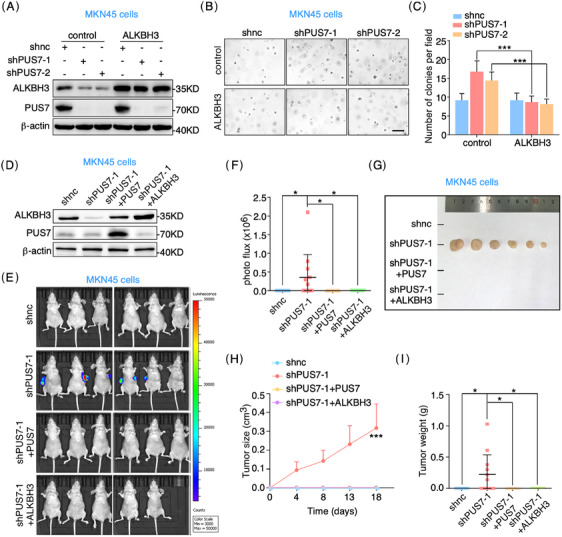
ALKBH3 reverses the phenotype induced by PUS7 depletion in gastric cancer model. (**A**–**C**) MKN45 cells treated with the indicated shRNAs were infected with lentivirus expressing ALKBH3 and subjected to western blots and 3D colony formation assays. Quantification of colony number per field is presented. Scale bar, 200 µm. (**D**–**I**) MKN45 cells treated with the indicated shRNAs were infected with lentivirus expressing PUS7 or ALKBH3 and applied for subcutaneous implantation in BALB/C nude mice (6 mice in each group, and one mouse in the shPUS7+ALKBH3 group died from a non‐tumour‐related bacterial infection before the experiment concluded). Tumour growth was detected by BLI analysis. The tumour growth curve (H) and weight (I) were shown. Data are expressed as means ± SD. Student's *t*‐test (tumour growth data (H) are expressed as means ± SEM. Two‐way ANOVA); *p < .05, ***p < .001.

Next, we performed rescue experiments to clarify if ALKBH3 mediates the function of PUS7 in gastric tumourigenesis. Our data displayed that ectopic expression of ALKBH3 significantly inhibited the proliferation phenotype induced by PUS7 depletion in MKN45 cells (Figure 5A–C). BLI experiments of the xenograft mouse model revealed that BLI signals were also increased in MKN45 cells depleted of PUS7, which was significantly reversed by ectopic expression of ALKBH3 or PUS7 (Figure 5D–F). Macroscopically subcutaneous tumours of PUS7‐depleted MKN45 cells were robustly diminished by exogenous expression of ALKBH3 or PUS7 (Figure 5G–I). Collectively, these findings indicate that PUS7 inhibits gastric tumour growth via ALKBH3.

### 3.6 PUS7 and ALKBH3 expression is significantly correlated in gastric cancer tissues

**FIGURE 6:**
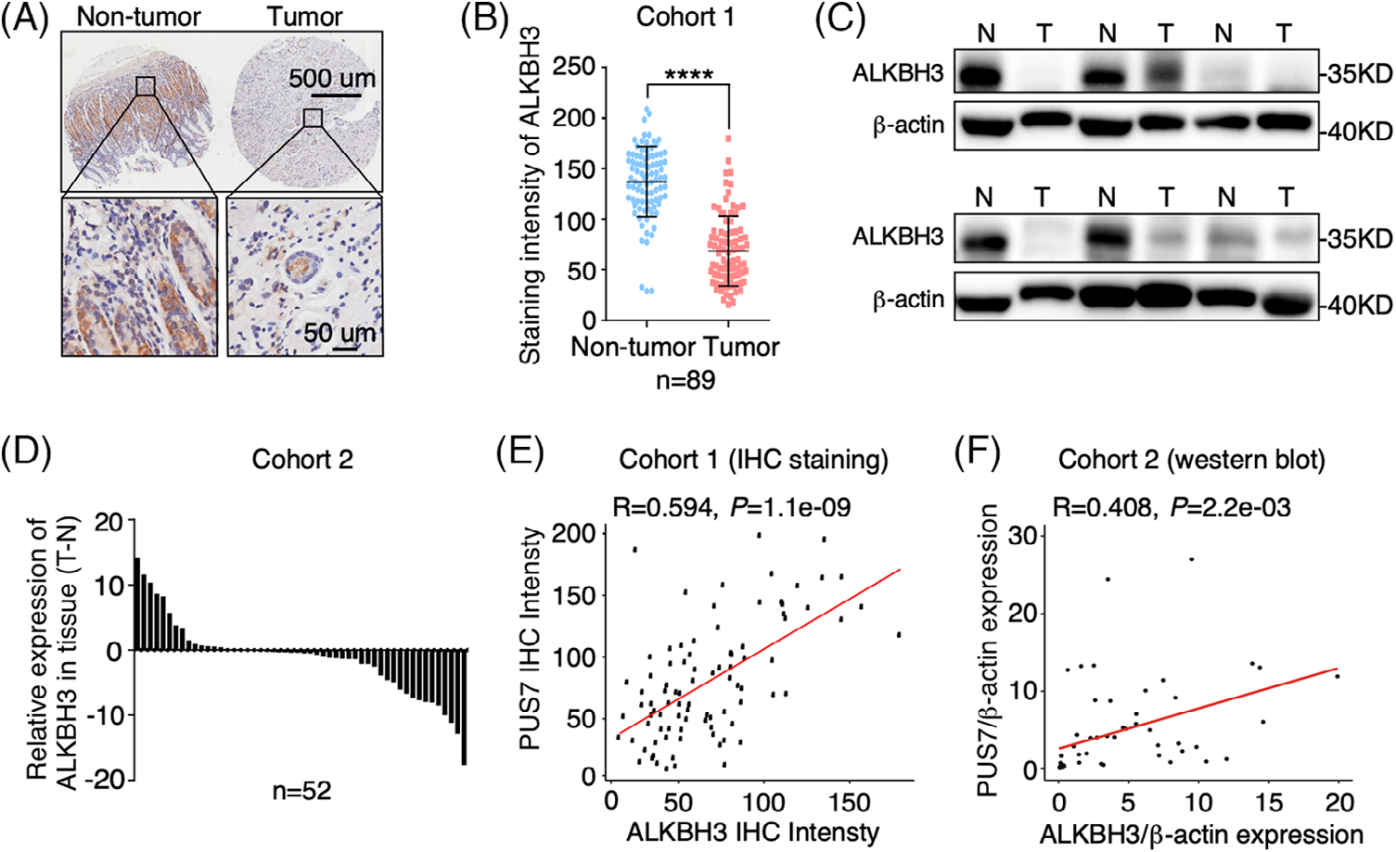
ALKBH3 expression is significantly decreased in gastric tumour tissues and correlated with PUS7 expression. (**A, B**) Immunohistochemistry analysis with anti‐ALKBH3 antibody in gastric tumour tissue array from cohort 1. Relative ALKBH3 expression was analyzed. Data are shown as mean ± SD. Student's *t*‐test; ****p < .0001. (**C, D**) Western blotting of gastric tumour tissues and their corresponding non‐tumour tissues with anti‐ALKBH3 antibody from cohort 2. β‐actin, a loading control. N, non‐tumour; T, tumour. The relative levels of ALKBH3 were quantified. (**E, F**) Pearson correlation analysis of PUS7 and ALKBH3 protein levels in gastric tumour tissues from two independent cohorts.

To evaluate the clinical association of PUS7 with ALKBH3 expression, we first examined the levels of ALKBH3 protein in human gastric tumour tissues and their paired non‐tumour tissues. Immunohistochemical analysis of tissue microarrays revealed a significant decrease in ALKBH3 expression in gastric tumour tissues from cohort 1 (Figures 6A and B and S8). Western blotting confirmed a noticeable decrease in ALKBH3 protein in gastric tumour tissues compared to their corresponding tissues from cohort 2 (Figure 6C and D). Furthermore, we carefully analyzed the association of PUS7 and ALKBH3 expression in gastric tumour tissues and found a significant correlation between PUS7 and ALKBH3 levels in gastric tumour tissues in both cohorts (Figure 6E and F).

## 4 DISCUSSION

**FIGURE 7:**
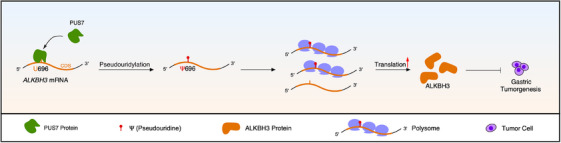
Schematic illustration shows that PUS7 modifies *ALKBH3* mRNA with pseudouridine (Ψ), enhancing its translation efficiency and suppressing gastric carcinogenesis.

Our study provides compelling evidence that both PUS7 and ALKBH3 are significantly and coordinately reduced in gastric tumour tissues, demonstrating their tumour‐suppressive function in gastric carcinogenesis. Mechanistically, PUS7 catalyzes the pseudouridylation of *ALKBH3* mRNA on the U696 site to enhance its translation efficiency and increase its protein levels, leading to the suppression of gastric tumour growth (Figure 7).

Previous research has documented that PUS7 has the capability to modify Ψ in both mRNAs and tRNAs within mammalian cells,^7^, ^8^, ^9^, ^12^, ^17^ highlighting the importance of investigating the biological function of PUS7‐mediated pseudouridylation. Most studies have focused on tRNA due to the difficulty of detecting Ψ on mRNA. For instance, PUS7's involvement in promoting glioblastoma stem cell tumourigenesis has been established through PUS7‐dependent tRNA modification.^7^ Additionally, PUS7 has been identified as a regulator of protein biosynthesis in embryonic stem cells by affecting tRNA‐derived small fragments (tRF).^17^ Our investigation has uncovered a compelling mechanism whereby PUS7 mediates mRNA pseudouridylation in the context of gastric cancer progression. Future studies are warranted to explore whether PUS7 also participates in regulating tRNA pseudouridylation and assess its impact on the progression of gastric cancer.

With the development of sequencing technology, high‐throughput profiling of Ψ on mRNA reveals that Ψ has a widespread and dynamic distribution with the potential to influence various cellular processes, such as pre‐mRNA splicing, RNA turnover, and mRNA translation.^9^, ^10^, ^11^, ^12^ However, the precise functional readout of pseudouridylated mRNA remains largely unclear. Our study sheds new light on this issue by demonstrating that PUS7‐dependent pseudouridylation of *ALKBH3* mRNA increases the translation efficiency of *ALKBH3* mRNA and subsequently suppresses gastric cancer cell proliferation and tumour growth. These observations provide functional evidence supporting the notion that mRNA pseudouridylation plays an indispensable role in cancer development.

PUS7 has been shown to have oncogenic functions in some tumours. For instance, PUS7 promotes glioblastoma progression through PUS7‐dependent tRNA pseudouridylation^7^, ^8^ and enhances the metastasis of colorectal cancer cells through a pseudouridylation‐independent mechanism.^18^ However, our data show that PUS7 expression is decreased in human gastric cancer tissues from two independent cohorts by using western blot and immunohistochemistry analyses. Furthermore, we find that PUS7 inhibits the proliferation and tumour growth of gastric cancer cells in a manner that depends on its catalytic activity. These data suggest that the function of PUS7 in cancer may be tissue‐specific and influenced by the complex nature of tumourigenesis and the unique tumour microenvironment.

Our findings show that ALKBH3 acts as a tumour suppressor in gastric cancer by inhibiting cell proliferation and tumour growth. Previous studies have found that ALKBH3 plays a role in repairing alkylation damage to DNA through oxidative demethylation of 1‐methyladenine (m^1^A) and 3‐methylcytosine (m^3^C) in single‐stranded DNA.^20^, ^31^ Additionally, ALKBH3 has been shown to play an important role in the demethylation of m^1^A and m^3^C in RNAs, which is essential for maintaining the stability and proper function of RNA molecules.^20^, ^32^ Further studies are needed to determine which role of ALKBH3 is responsible for its regulatory effect on gastric tumour growth.

Gastric cancer was used as the model in this study to elucidate the role of PUS7‐ALKBH3 pseudouridylation axis in cancer. It is unknown whether ALKBH3 can function as a substrate for PUS7 in other cancer types. Despite the very different responses to genes in different cancer cells,^33^ further validation is needed to explore the specificity of PUS7‐ALKBH3 pseudouridylation axis in gastric cancer.

Previous studies have shown that replacing uridines with pseudouridines in transcribed mRNA enhances translation. However, the underlying reasons for this enhancement have only been partially explored in vitro.^34^, ^35^ Our analysis revealed that PUS7 deletion reduces the binding of *ALKBH3* mRNA to the ribosome, which may be one of the reasons why PUS7‐mediated *ALKBH3* mRNA pseudouridylation promotes its translation. The regulatory mechanisms of PUS7‐mediated *ALKBH3* mRNA pseudouridylation on protein translation using both biological and chemical methods will be a focus of our future research.

## 5 CONCLUSIONS

Our study demonstrates that the expression levels of both PUS7 and ALKBH3 are significantly diminished in gastric cancer tissues. PUS7 impacts gastric cancer cells by inhibiting their proliferation and tumour growth through its Ψ catalytic activity. Crucially, PUS7 modifies *ALKBH3* mRNA with pseudouridine (Ψ), enhancing its translation efficiency and consequently suppressing gastric tumour growth. These findings illuminate the pivotal role of PUS7‐dependent pseudouridylation of *ALKBH3* mRNA in hindering gastric carcinogenesis.

## AUTHOR CONTRIBUTIONS

Conceptualisation, T.Z. and S.X. Methodology: S.X. and Y.C. Investigation, Y.C., H.J., Y.C., K.C., W.K., C.H., Z.X. and Y.L. Visualisation, Y.C., F.Y., Y.C., and H.J. Writing – original draft, S.X. and Y.C. Writing – review & editing, T.Z., S.X., A.L., B. Y. and W.L. Supervision, T.Z. and S.X.

## CONFLICT OF INTEREST STATEMENT

The authors declare they have no conflicts of interest.

## FUNDING

This work was supported by the National Key Research and Development Program of China (2019YFA0802202), the Fundamental Research Funds for the Central Universities (491040*17221102401), and the National Natural Science Foundation of China (U21A20197 and 32270723).

## ETHICS STATEMENT

The study was approved by Ethics Committee Review Board of Zhejiang University School of Medicine (Approval No. 2020−655), and mouse experiments were conducted in accordance with the Guide for the Care and Use of Animals for research purposes and were approved by the Committee of Animal Ethics, Zhejiang University (Approval No. ZJU20230035).

## Supporting information

Supporting Information

## Data Availability

This paper does not report original code. Any additional information required to reanalyze the data reported in this paper is available from the lead contact upon request.
