# Supplementary material for: PUS7‐dependent pseudouridylation of ALKBH3 mRNA inhibits gastric cancer progression
Source: Clin Transl Med. 2024 Aug 23;14(8):e1811. doi: 10.1002/ctm2.1811 (PMC11341916; doi:10.1002/ctm2.1811)
Supplement: Supplementary file 1 — Supporting Information [file CTM2-14-e1811-s001.docx]

**Supplementary figures**

**
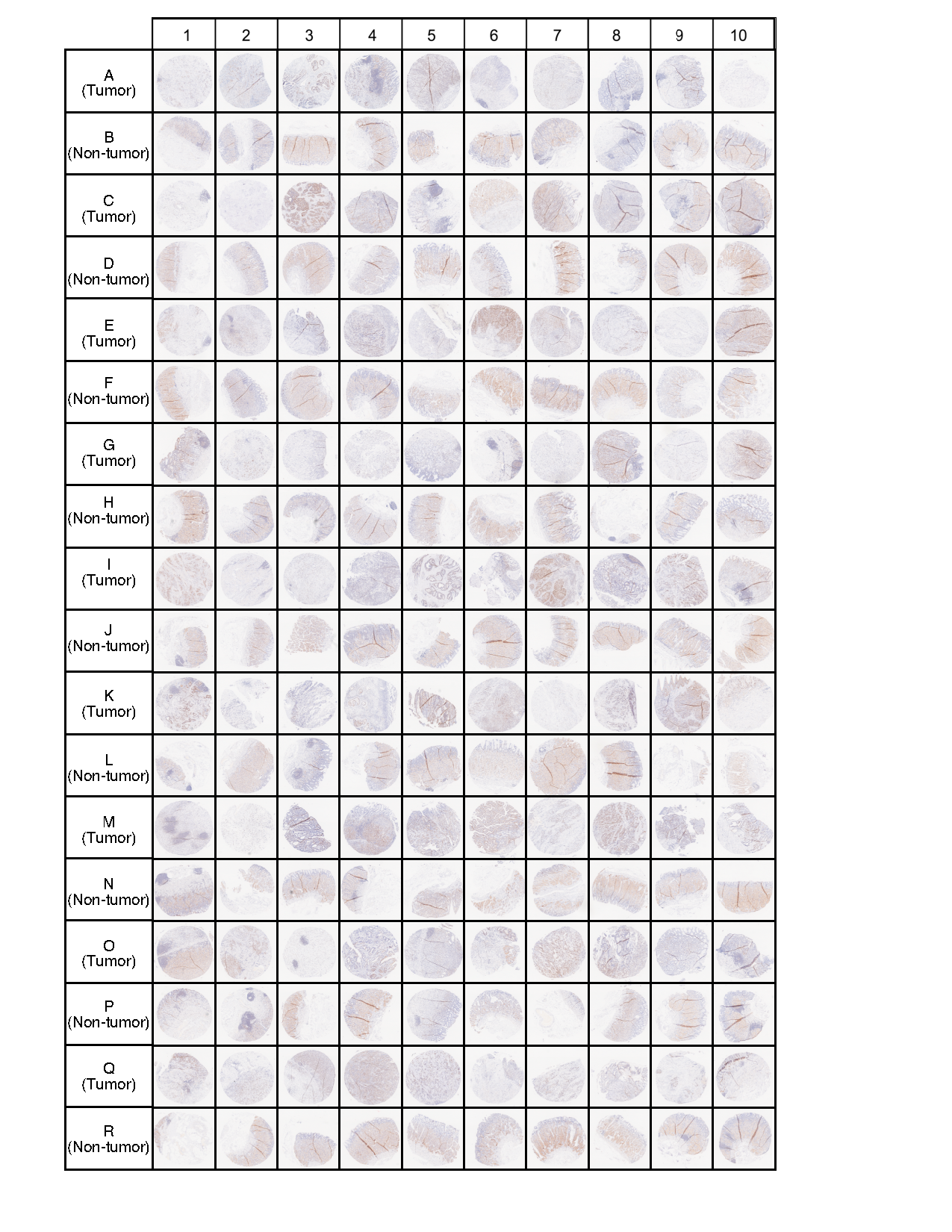
**

**Figure S1.** The overall image of the gastric cancer tissue microarrays from cohort 1 after immunohistochemical staining with anti-PUS7 antibody.


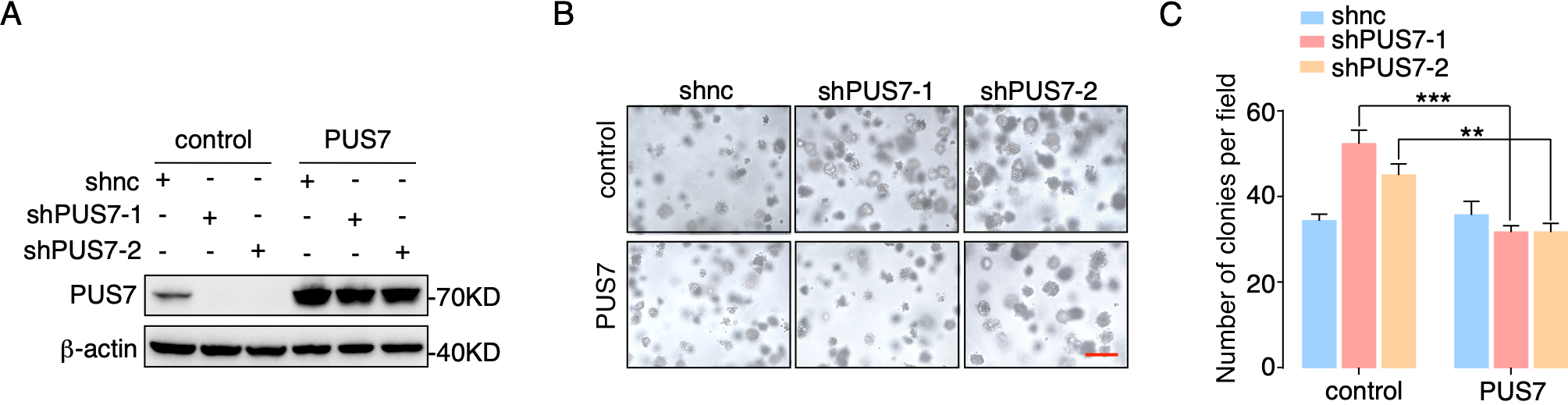


Figure S2. PUS7 attenuates the enhanced proliferation ability of PUS7-depleted cells

A-C, MKN45 cells treated with the indicated shRNAs were infected with the lentivirus expressing PUS7, and subjected to western blots and 3D colony formation assays. Quantification of colony numbers is shown. Scale bar, 200 µm. Data are expressed as means ± SD. Student’s *t* test; ***P* < 0.01, ****P* < 0.001.


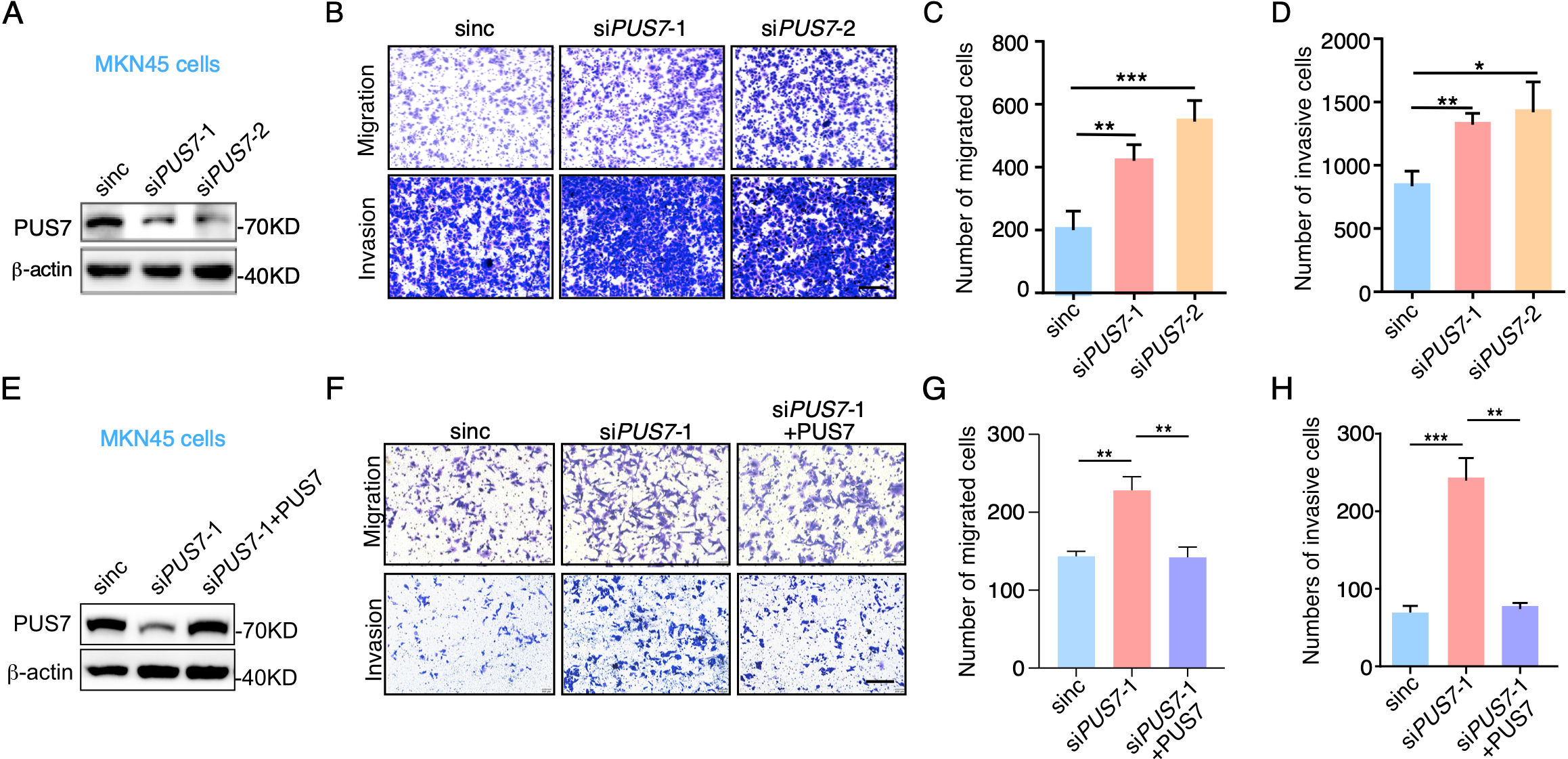


Figure S3. Depletion of PUS7 promoted migration and invasion of gastric cancer cells

A-D, MKN45 cells treated with small interfering RNAs (siRNAs) targeting PUS7 were subjected to western blots, migration and invasion assays. E-H, MKN45 cells treated with the indicated siRNAs were infected with the lentivirus expressing PUS7, and subjected to western blots, migration and invasion assays. Quantification of migrated (C, G) and invasive (D, H) cell numbers is shown. Scale bar, 200 µm. Data are expressed as means ± SD. Student’s *t* test; **P* < 0.05, ***P* < 0.01, ****P* < 0.001.


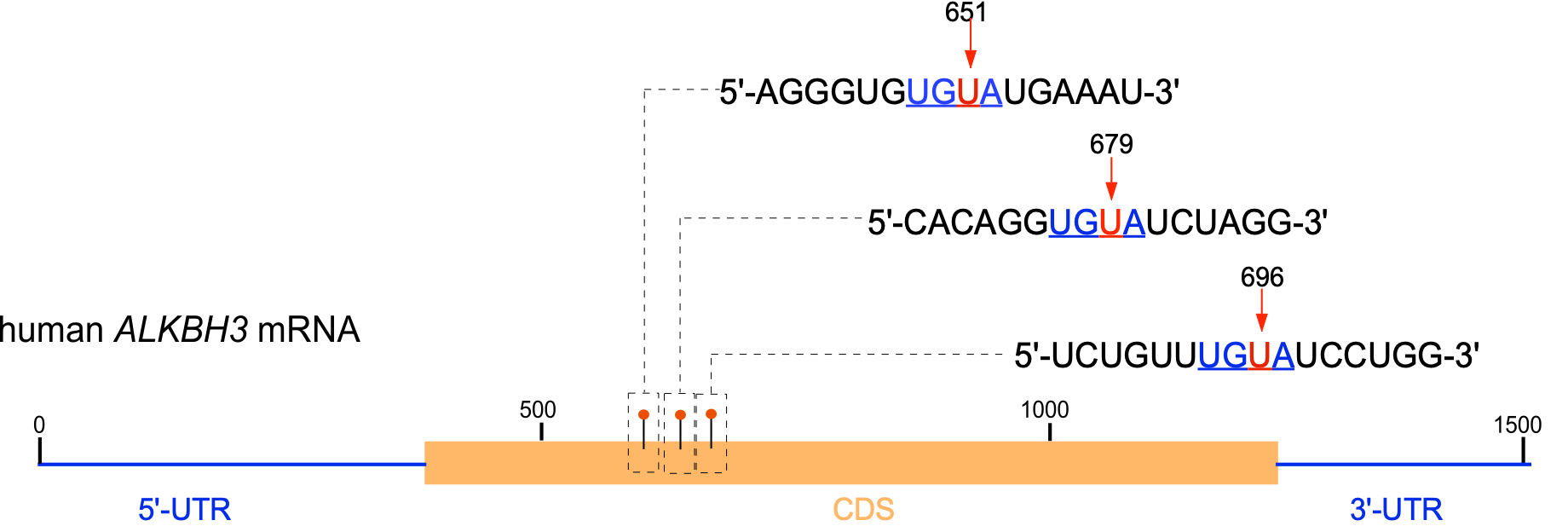


Figure S4. Schematic of three putative Ψ consensus motifs on *ALKBH3* mRNA

Three potential sites (U651, U679 and U696) on *ALKBH3* mRNA according to PUS7 core consensus sequence for pseudouridylation (UGUA, underline for Ψ site).


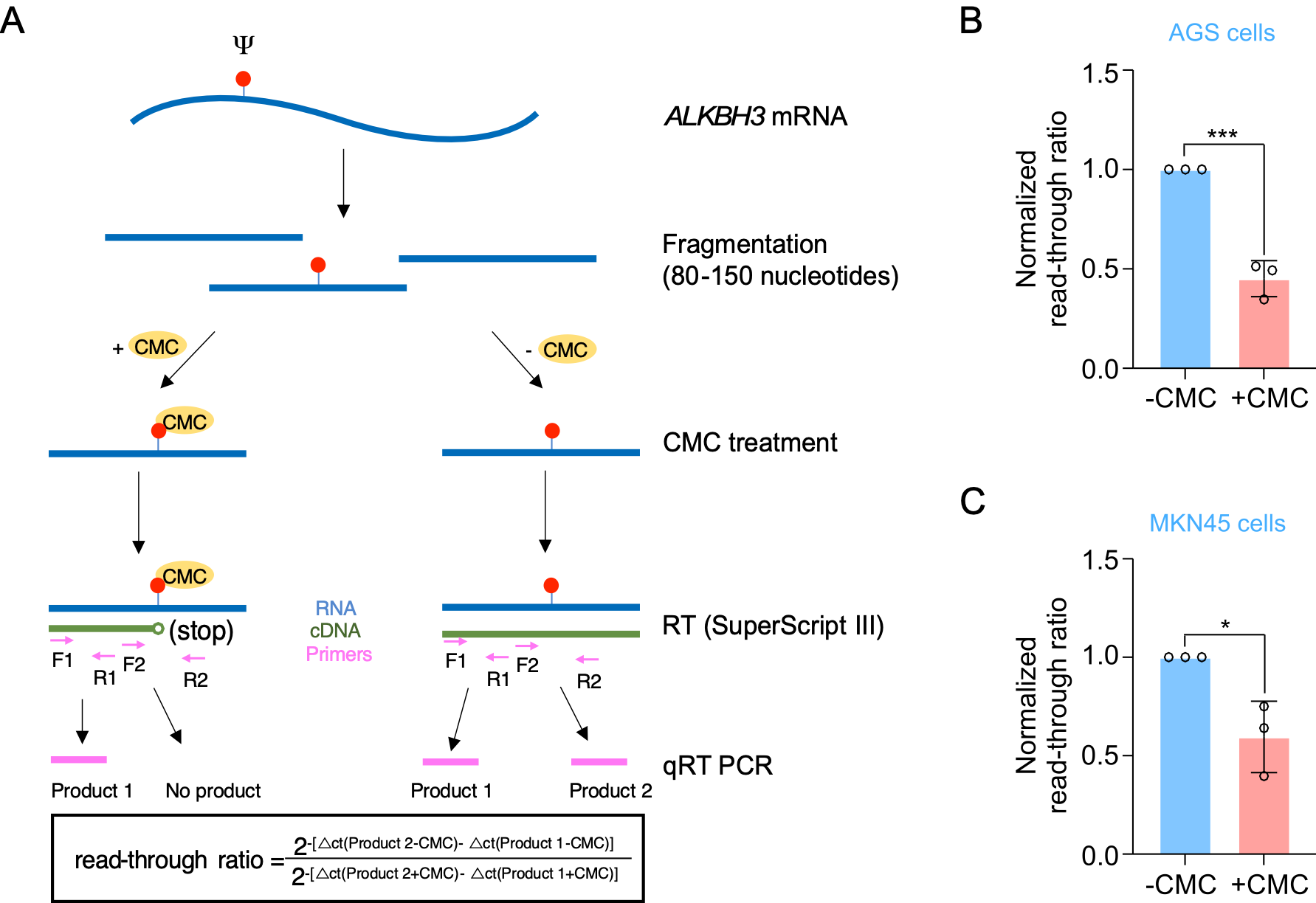


Figure S5. Validation of the Ψ sites on *ALKBH3* mRNA

A, A flowchart of CMC-based RT-qPCR assay to quantify read-through ratios (the CMC-modified Ψ blocks RT and the following qPCR amplification). B, C, Readthrough ratio at Ψ sites on *ALKBH3* mRNA of CMC-based RT-qPCR assay as shown. Data are expressed as means ± SD. Student’s t test; **P* < 0.05, ****P* < 0.001.


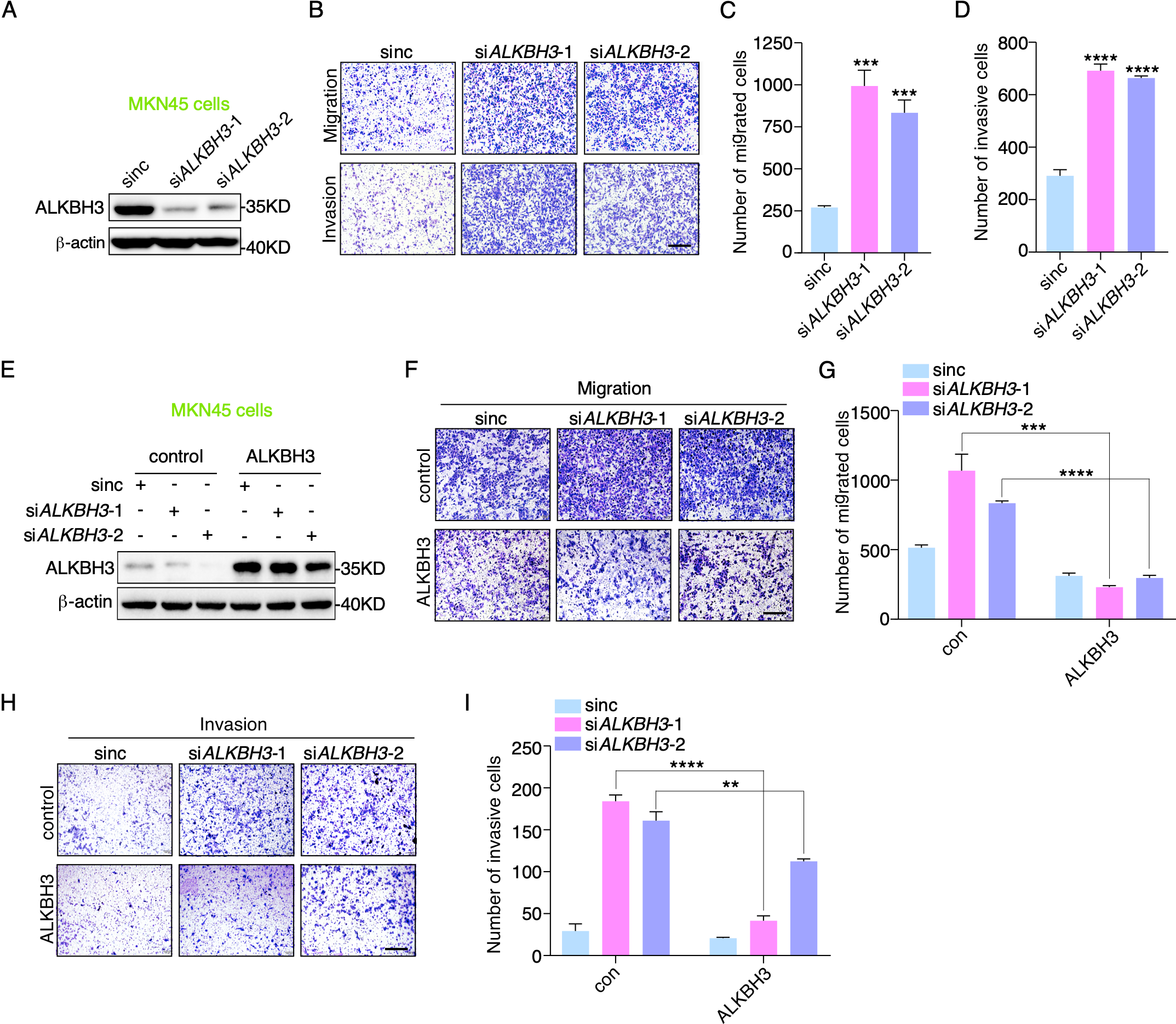


Figure S6. Depletion of ALKBH3 promoted migration and invasion of gastric cancer cells

A-D, MKN45 cells treated with siRNAs targeting ALKBH3 were subjected to western blots, migration and invasion assays. E-I, MKN45 cells treated with the indicated siRNAs were infected with the lentivirus expressing ALKBH3, and subjected to western blots, migration and invasion assays. Quantification of migrated (C, G) and invasive (D, I) cell numbers is shown. Scale bar, 200 µm. Data are expressed as means ± SD. Student’s *t* test; **P* < 0.05, ***P* < 0.01, ****P* < 0.001.


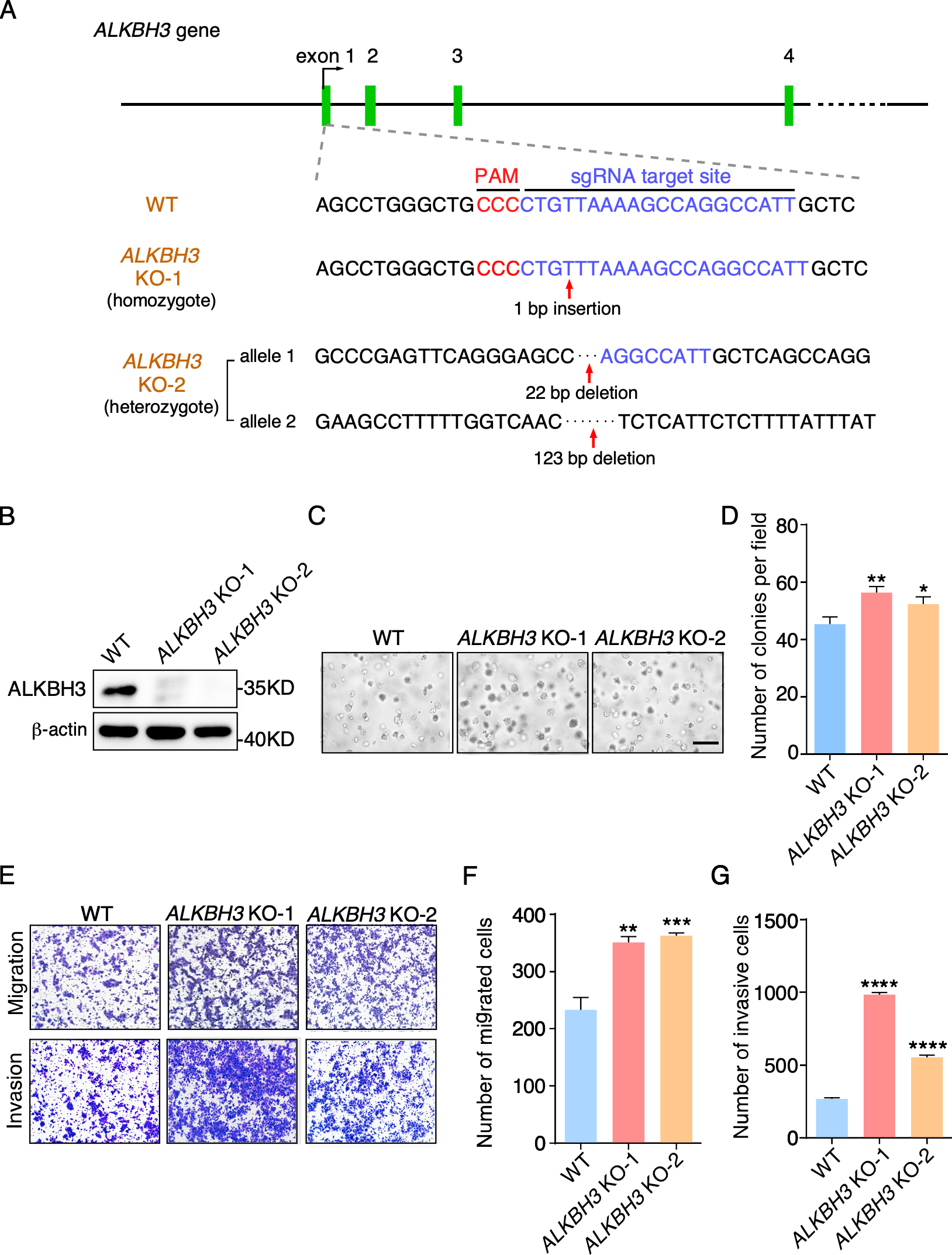


Figure S7. ALKBH3 deletion promotes gastric cancer cell proliferation

A, The sequence of indels in the *ALKBH3* locus of MKN45 cells generated by the CRISPR/Cas9 system is presented. The PAM and sgRNA target sites are indicated in red and blue, respectively. B-G, Wild-type or *ALKBH3* knockout MKN45 cells were applied for western blots analysis with anti-ALKBH3 antibody (B), 3D colony formation (C, D), migration and invasion (E-G) assays. β-actin, a loading control. Quantification of colony (D), migrated (F) and invasive (G) cell numbers per field is shown. Scale bar, 200 µm. Data are expressed as means ± SD. Student’s *t* test; **P* < 0.05, ***P* < 0.01, ****P* < 0.001, *****P* < 0.0001.


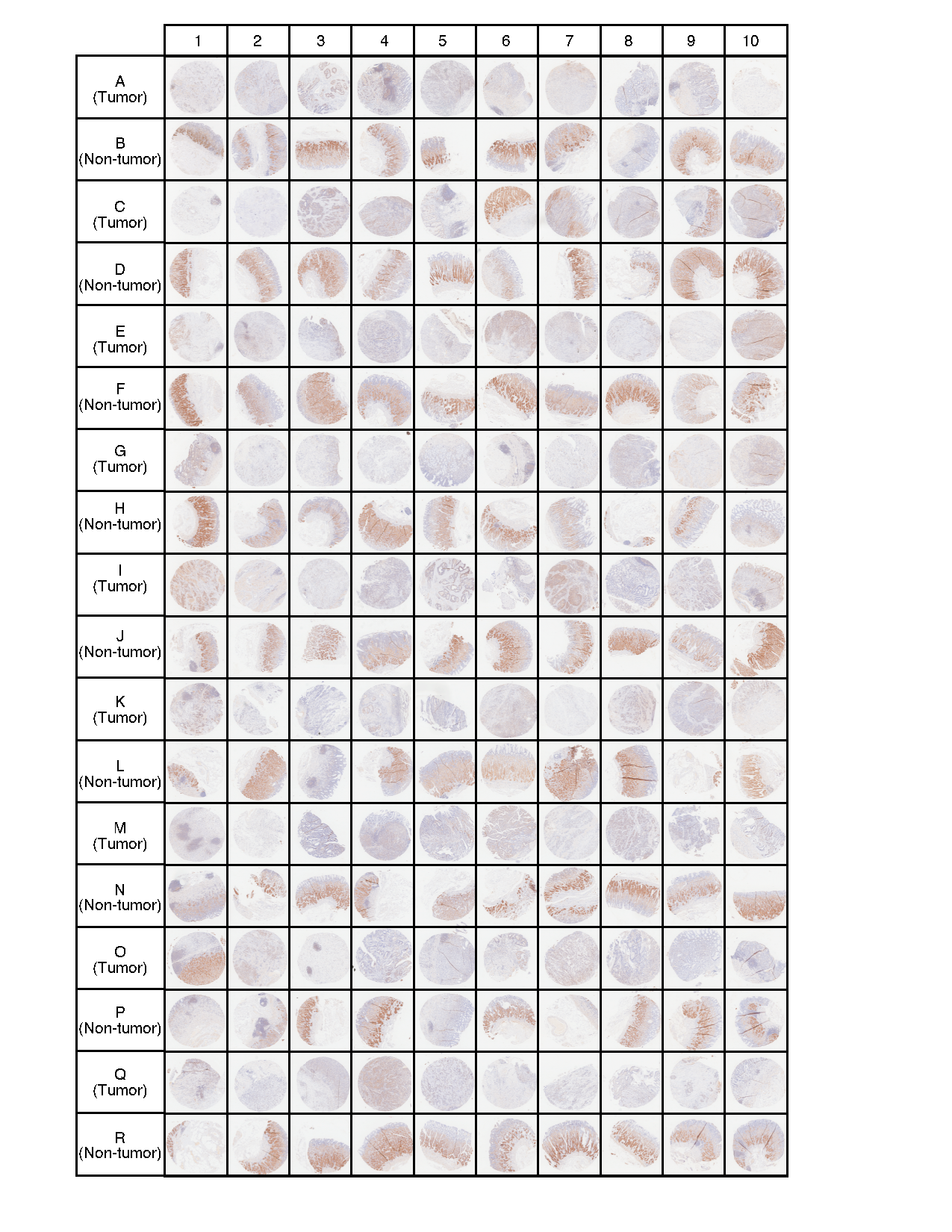


**Figure S8.** The overall image of the gastric cancer tissue microarrays from cohort 1 after immunohistochemical staining with anti-ALKBH3 antibody.

Supplementary Tables

Table S1. The primers used for plasmid construction

| **Vectors** | **Primers** | **Sequences** |
| --- | --- | --- |
| Flag-PUS7 | Forward | GTCACTCGAGATGGACTACAAAGACGAT GACGACAAGGACGAGATGACAGAAAT |
|  | Reverse | GTCAGGATCCTCAGCGAAGCCAGGTTGTAT |
| PUS7-D294A | Forward | GGAACCAAAGCTAAAAGGGCTATAACAG |
|  | Reverse | CCCTTTTAGCTTTGGTTCCCATGTAGGA |
| ALKBH3 | Forward | GTCAGAATTCATGGAGGAAAAAAGACGGCG |
|  | Reverse | GTCAGGATCCTCACCAGGGTGCCCCTCGAG |

Table S2. The sgRNAs sequences

| **Gene name** | **Primers** | **Sequences** |
| --- | --- | --- |
| PUS7 | Forward | CACCGACTGGTGTGTCGCTGAAACG |
|  | Reverse | AAACCGTTTCAGCGACACACCAGTC |
| ALKBH3 | Forward | CACCGATGGCCTGGCTTTTAACAG |
|  | Reverse | AAACCTGTTAAAAGCCAGGCCATC |

Table S3. The primers used for RT-qPCR

| **Gene name** | **Primers No.** | **Primers** | **Sequences** |
| --- | --- | --- | --- |
| PUS7 | 1# | Forward | ACAGAGGATAGGGAGGGGAA |
|  |  | Reverse | CTGCAGTTCTGACCTTTGCC |
| ALKBH3 | 1# | Forward | ACTAGGGAGGTGCCCCATTA |
|  |  | Reverse | TCTCTTCTGGTGGTGGCTTCT |
|  | 2# | Forward | AGAACCTCAGCAGGTAGT |
|  |  | Reverse | TATATCCTCTCTGATGCC |
| U6 | 1# | Forward | CTCGCTTCGGCAGCACA |
|  |  | Reverse | AACGCTTCACGAATTTGCGT |
